# Total saponins from *Albizia julibrissin* inhibit vascular endothelial growth factor-mediated angiogenesis *in vitro* and *in vivo*

**DOI:** 10.3892/mmr.2015.3228

**Published:** 2015-01-20

**Authors:** WEIWEI CAI, YUE LI, QINGQING YI, FENGSHAN XIE, BIN DU, LEI FENG, LIYING QIU

**Affiliations:** 1 Laboratory of Natural Medicine, School of Pharmaceutical Science, Jiangnan University, Wuxi, Jiangsu 214122, P.R. China; 2 Laboratory of Tumor Pharmacology, Wuxi Medical School, Jiangnan University, Wuxi, Jiangsu 214122, P.R. China

**Keywords:** TSAJ, angiogenesis, VEGF, Ea.hy926 cells, Matrigel™ plug

## Abstract

Dried stem bark from *Albizia julibrissin* (AJ) is a highly valued Traditional Chinese Medicine, which has been shown to suppress tumor growth and angiogenesis. Total saponins from AJ (TSAJ) are one of the most bioactive components of AJ extract. The present study evaluated the anti-tumor and anti-angiogenic effects of TSAJ *in vitro* and *in vivo*. The anti-angiogenic activity of TSAJ was investigated by measuring the effects on vascular endothelial growth factor (VEGF)-induced proliferation, migration and tube formation of Ea.hy926 endothelial cells *in vitro*. The expression levels of proteins associated with VEGF-induced angiogenesis were determined by western blotting. Furthermore, *in vivo* Matrigel™ plug and H22 hepatoma tumor models were used to verify the anti-angiogenic effects of TSAJ. The present study demonstrated that TSAJ significantly inhibited VEGF-mediated endothelial cell proliferation, migration and tube formation of Ea.hy926 cells *in vitro*. The anti-angiogenic effects of TSAJ were modulated by suppression of phosphorylated-(p-) focal adhesion kinase, p-Akt, and p-extracellular signal-regulated kinase in the VEGF/VEGF receptor 2 (R2) signaling pathway. Furthermore, oral administration of TSAJ significantly inhibited tumor growth and tumor-induced angiogenesis, as well as the formation of functional vessels, in the Matrigel™ plug model. These results suggest that TSAJ may be a potential anti-angiogenic agent that targets the VEGF/VEGFR2 signaling pathway, and inhibits tumor-induced angiogenesis.

## Introduction

The concept that ‘tumor growth is angiogenesis-dependent’ was initially proposed by Folkman in 1971 (1), and suggests that the growth and metastasis of tumors depend on the development of blood vessels. In the early stages of cancer, the uncontrolled proliferation of cancer cells leads to a shortage of nutrients and oxygen, which causes a large degree of cell death (2,3). In response to the abnormal microenvironment, angiogenic molecules secreted by the tumor stimulate the formation of new functional vessels, from the pre-existing vasculature (4). Once the tumor cells acquire the ability to induce angiogenesis, tumor expansion is initiated, and tends to be malignant (5). Therefore, inhibiting the angiogenic process may be used as a potential therapy to prevent tumor growth and metastasis. Among the angiogenic factors, vascular endothelial growth factor-A165 (VEGF-A165, commonly referred to as VEGF) is positively associated with the proliferation, migration and tube formation of endothelial cells (6,7).

Upon VEGF binding to VEGF receptor 2 (R2), the main functional receptor tyrosine kinases of VEGF are activated, and a cascade of events are initiated, including the phosphoinositide 3-kinase (PI3K)/protein kinase B (Akt) and RAF/MEK/ERK signaling pathways, which stimulate endothelial cell proliferation, migration and tube formation (8). Numerous studies have demonstrated that focal adhesion kinase (FAK), and its substrate paxillin, are involved in the signal transduction that participates in focal adhesion during cell migration (9–11).

*Albizia julibrissin* (AJ) (*Leguminosae*) is a Traditional Chinese Medicine that was recorded as an anti-inflammatory and sedative drug to treat swelling and pain in the lungs, shin ulcers, wounds and for the removal of carbuncles (12). In modern pharmacology, AJ exhibits marked inhibitory activity against certain cancer cell lines *in vitro* (13,14), which suggested that it may be used as an anti-tumor agent. In our previous study it was demonstrated that the crude extract of AJ exhibits anti-angiogenic effects on 3B11 and HMEC-1 cells (15). Further studies focused on the anti-angiogenic effects of AJ revealed that the active ingredients are the saponins. The total saponins from AJ (TSAJ), whose major constituents include julibroside J, julibroside A, julibroside B1, julibroside C1, julibroside I, julibroside II and julibroside III (16–20), have been shown to possess anti-angiogenic and anti-tumor activities; however, the underlying mechanisms of action remain to be elucidated. The present study investigated the anti-angiogenic effects of TSAJ on VEGF-induced angiogenesis *in vitro* and *in vivo*, in order to explore the potential for TSAJ as an anti-tumor drug targeting the VEGF/VEGFR2 signaling pathway.

## Materials and methods

### Preparation of TSAJ

Dried stem barks from AJ (lot no. 20100154) were obtained from Zhejiang (China) and were identified by Professor Jian-wei Chen (Chinese Herbal Pharmacy, Nanjing University of Traditional Chinese Medicine, Nanjing, China). The voucher specimens were maintained in the herbarium stock room at the Laboratory of Natural Medicine, School of Pharmaceutical Science, Jiangnan University (Wuxi, China).

**Figure 1:**
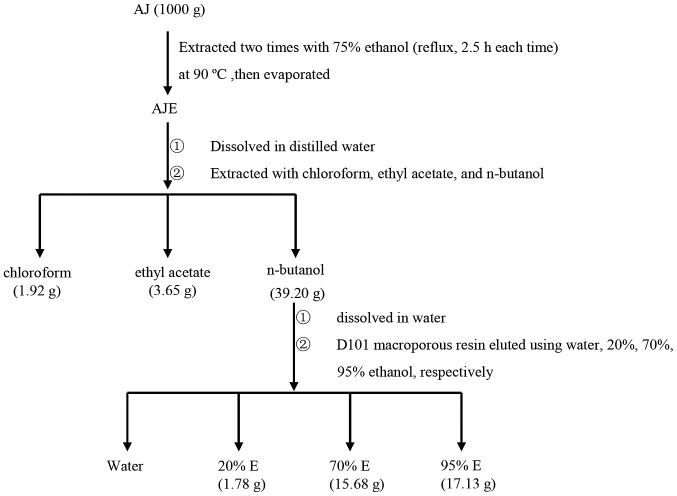
Preparation of total saponins of *Albizia julibrissin* (AJ) isolated from AJ extracts (AJE). E, ethanol.

The TSAJ sample used in the present study was originally isolated from the AJ specimens. Briefly, the powder of AJ dried stem barks was extracted twice with 75% ethanol (reflux, 2.5 h each time) at 90°C. The ethanol extract was then evaporated, dissolved in deionized water and sequentially extracted with chloroform, ethyl acetate and *n*-butanol. The *n*-butanol fraction, which contained the most bioactive components, was dissolved in water and subjected to D101 macroporous resin (Tianjin Bohong Resin Technology, Tianjin, China) to yield 20% ethanol (20% E), 70% ethanol (70% E), 95% ethanol (95% E), and water components (Fig. 1).

### Preliminary phytochemical screening of total saponins

The presence of saponins in the four fractions was assessed using foam and hemolytic tests.

### Foam test

The four dried fractions (10 mg) were placed in a graduated cylinder with 10 ml distilled water. The suspension was shaken for 30 sec and a 2–3 cm layer of foam indicated the presence of saponins.

### Hemolytic test

Once the hemaleucin in the anticoagulant whole blood of rabbit was discarded, the blood was washed three times with 0.9% normal sodium solution. Subsequently, the prepared erythrocytes were suspended in 0.9% saline solution to a final concentration of 2% (v/v). The solutions of the four fractions (1 ml) were separately added to the various erythrocyte suspensions (3 ml), and incubated at 37°C for 1 h. Normal sodium solution (0.9%) was used as a control. The hemolytic degree of the four fractions was assayed by observation. Briefly, following incubation, the mixtures were then centrifuged at room temperature for 5 min at 495 × g to separate the supernatant (hemoglobin) and the precipitation (complete erythrocytes and cell debris). If the solution in the tube was transparent and red, significant hemolysis phenomena was shown, thus it indicated the presence of saponins. If the supernatant was transparent and colorless, and all erythrocytes were sunk, thus it indicated the absence of saponins (21).

### Antibodies and other materials

Human VEGF-A_165_ was obtained from PeproTech, Inc. (Rocky Hill, NJ, USA). Matrigel™ was obtained from BD Biosciences (Franklin Lakes, NJ, USA). Rabbit polyclonal antibodies targeting β-tubulin (2148S), p^Tyr1175^-VEGFR2 (2478S), p^Tyr576/Tyr577^-Fak (3281S), p^Ser473^-Akt (8200S) and p^Thr202/Thr204^-extracellular signal-regulated kinase (Erk)1/2 (4370S) were purchased from Cell Signaling Technology (Danvers, MA, USA). Cluster of differentiation 31 (CD31) was purchased from Sangon Biotech Co., Ltd (Shanghai, China). Goat anti-rabbit immunoglobulin G (IgG) (H+L) horseradish peroxidase (HRP)-conjugated antibodies (21621) were purchased from EMD Millipore (Billerica, MA, USA). Reagents, including ethanol, chloroform, ethyl acetate, *n*-butanol, formalin and paraffin were purchased from China National Medicines Corporation (Beijing, China). Cell Counting kit-8 (CCK-8) was purchased from Beyotime Institute of Biotechnology (Haimen, China).

### Animals

Female BALB/c mice (17–20 g) were purchased from the Research Center of Laboratory Animals (Hangzhou, China; grade specific-pathogen free and certificate no. SCXK (Zhe) 2008-0033). The mice were maintained in a barrier facility, in a temperature- and humidity-controlled environment, fed sterilized food and were given *ad libitum* access to water. Prior to the experiment, all of the mice were allowed to acclimate for one week. All experiments were conducted according to the Guides for the Care and Use of Laboratory Animals (Ministry of Science and Technology of China, 2006), and were approved by the Animal Ethics Committees of Jiangnan University (JN NO 20130327-0702).

### Cell culture

The Ea.hy926 human endothelial cell line, generated from fusion of the A549 epithelial cell line with HUVEC, was provided by Professor Quan-sheng Zhou (Soochow University, Suzhou, China). The cells were cultured in cell medium consisting of Dulbecco’s Modified Eagle Medium (DMEM; Hyclone Laboratories, Inc., Logan, UT, USA) supplemented with 10% (v/v) fetal bovine serum (FBS; Gibco Life Technologies, Carlsbad, CA, USA), in 25 cm^2^ culture flasks at 37°C in an atmosphere containing 5% CO_2_.

### Determination of anti-angiogenic effects in vitro

#### Cell proliferation assay

The viability of the Ea.hy926 cells was assessed using a CCK-8. Briefly, the Ea.hy926 cells (6×10^3^ cells/well) were plated in 96-well plates (Corning, Inc., Corning, NY, USA) and cultured in normal growth medium for 24 h. The culture medium was then replaced with normal growth medium containing various concentrations of TSAJ (0, 0.78125, 1.5625, 3.125, 6.25, 12.5, 25, and 50 μg/ml), for 12, 24 and 48 h. Subsequently, the medium was replaced with DMEM containing 10% CCK-8. Following a 2 h incubation at 37°C, the absorbance of the resulting product was measured at a wavelength of 450 nm, using an ELISA microplate reader (Thermo Labsystems, Waltham, MA, USA). The percentage viability of the cells was then calculated using the following formula: viability (% of control) = (OD_control_-OD_treated_)/OD_control_ ODcontrol and ODtreated represent the average OD450 value of cells in the control and TSAJ-treated groups, respectively. Three independent experiments were performed.

The effects of TSAJ on VEGF-induced cell viability were determined as described by previous methods (22). Briefly, the Ea.hy926 cells (6×10^3^ cells/well) were seeded in 96-well cell plates and cultured in normal growth medium for 24 h. Subsequently, the cells were exposed to various concentrations of TSAJ (0, 0.78125, 1.5625, 3.125, 6.25, 12.5, 25, and 50 μg/ml), with or without VEGF (10 ng/ml), for 48 h in DMEM supplemented with 5% FBS. The medium was then replaced with DMEM containing 10% CCK-8. Following a 2 h incubation at 37°C, the absorbance of the resulting product was measured at 450 nm, using an ELISA microplate reader (Labsystem, USA). The percentage viability of the cells was then calculated. At least three independent experiments were performed. The control group, which did not receive VEGF or TSAJ treatment, was set at 100%.

#### Migration activity assay

The migratory ability of the cells was assessed using a scratch-wound directional assay. Briefly, the Ea.hy926 cells were seeded at a cell density of 8×10^4^ cells/well in a 24-well cell plate (Corning, Inc.), and grown overnight into a confluent monolayer. A sterile 20–200 μl micropipette tip (Axygen^®^; Corning Life Sciences, Tewksbury, MA, USA) was then used to create a ‘wound field’ of ±1000 μm width. The cells were washed twice with phosphate-buffered saline (Boster, Wuhan, China) and replaced in fresh medium containing the indicated concentrations of TSAJ, supplemented with 0.5% FBS and VEGF (10 ng/ml).

Following a 14 h incubation, the migrated cells were photographed using an inverted microscope (TE2000-S; Nikon Corporation, Tokyo, Japan) with NIS-Elements software. Cell migration was estimated by measuring the endothelial cells that had migrated from the edge of the wounded monolayer (23). The percentage of migration was the mean calculated from five replicates of each experiment. Three independent experiments were performed. The control group, which did not receive VEGF or TSAJ treatment, was set at 100%.

#### Tube formation assay

The effects of TSAJ on the morphogenesis of endothelial cells were investigated using a capillary tube formation assay on Matrigel™. Briefly, the Matrigel™ was thawed at 4°C overnight, and 80 μl was then added to a 96-well plate. Following a 45 min incubation at 37°C, a cell density of 4×10^4^ cells/well was seeded onto the Matrigel™-pre-coated 96-well plate. The cells were treated with TSAJ at 4.5 and 9 μg/ml, with or without VEGF (10 ng/ml). Following 6 h, tube formation was visualized and images were captured using an inverted microscope (TE2000-S; Nikon Corporation). Tube formation was quantified by counting the number of tubular structures within the capillary networks of five random fields (control group was set at 100%) using Image-Pro Plus 6.0 software (Media Cybernetics, Inc., Rockville, MD, USA). The results were the means calculated from five replicates of each experiment.

### Western blot analysis

Protein expression levels were analyzed by western blot analysis, as previously described (24). Briefly, 25 μg of total protein/well was loaded, following denaturing in loading buffer at 100°C for 5 min. The protein extracts were then separated by 8–12% SDS-PAGE and electrophoretically transferred to nitrocellulose membranes (EMD Millipore). Subsequently, the membranes were blocked at room temperature for 2 h in 5% nonfat dry milk/Tris-buffered saline with Tween^®^ (TBST). The membranes were then incubated at 4°C overnight with various primary antibodies. The primary antibodies used in the present study were as follows: p-VEGFR2 (1:2,000), p-Fak (1:2,000), p-Akt (1:1,000), p-Erk (1:2,000) and β-tubulin (1:1,000). The following day, the membranes were washed three times with TBST for 5 min at room temperature, and subsequently incubated with HRP-conjugated anti-rabbit IgG secondary antibodies, for 2 h at room temperature. Following the incubation, the membranes were washed with TBST, and the proteins bands were visualized using the Diaminobenzidine Detection system (Boster). β-tubulin was used as the protein loading control.

### Anti-angiogenic effects in vivo

#### In vivo Matrigel™ plug assay

A Matrigel™ plug assay was performed in BALB/c mice, as described previously with some modifications (25). Briefly, female BALB/c mice (5–6 weeks old; weighing 17–20 g), were subcutaneously injected with 500 μl Matrigel™ containing heparin (130 U; Sigma-Aldrich) and mouse VEGF (250 ng). Mice injected with Matrigel™ and heparin alone were included as a vehicle control. The following day, the mice were orally administrated a single dose of saline, or TSAJ at 1.8 or 3.6 mg/kg/d. Each group contained six mice and each dose was administered daily by oral gavage. After 14 days, the Matrigel™ plugs were carefully removed, photographed, formalin-fixed and paraffin-embedded.

To assay the microvascular density (MVD) of each group, Masson’s Trichrome (M-T; Nanjing Jiancheng Bioengineering Institute, Nanjing, China) staining was performed to visualize endothelial infiltration. Briefly, 3 μm sections were stained with M-T solution. The number of blood vessels in a high power field were quantified by counting vessel infiltration into the plugs.

#### In vivo anti-tumor effects

H22 hepatoma cells [2×10^6^ cells in 0.2 ml normal saline (NS)] were subcutaneously injected into the armpits of the right forearms of the mice. Once the tumor model had been established, the mice were randomly divided into three groups each containing 10 mice. The TSAJ-treated groups were orally administrated 1.8 mg/kg/d or 3.6 mg/kg/d of TSAJ. The vehicle control group was orally treated with the equivalent of 0.9% normal saline. Each dose was administered daily by oral gavage and the treatment lasted for 14 days. At the end of the experiment all the mice were sacrificed with an overdose of 10% chloral hydrate by intraperitoneal injection (400 mg/kg; Sigma-Aldrich), and the tumor tissue was segregated, weighed and photographed. The tumor inhibitory ratio was calculated using the following formula: Tumor inhibitory rate (%)=(*W*_control_-*W*_treated_)/*W*_control_ × 100%. *W*_control_ and *W*_treated_ represent the average weights of the tumor in the vehicle control and TSAJ-treated groups, respectively.

The tumor samples were then formalin-fixed and paraffin-embedded. The sections (4 μm) were stained with hematoxylin and eosin (KeyGen BioTech, Nanjng, China), or immunostained with antibodies targeting mouse CD31 (1:100), p-ERK (1:200), p-AKT (1:200), and p-VEGFR2 (1:200). The immunostaining procedures were performed according to the manufacturer’s instructions. MVD was calculated using Image-Pro Plus 6.0 software.

### Statistical analysis

All experiments were repeated three times. Data represent the mean ± standard error of the mean. Statistical significance was analyzed by unpaired Student’s t-test or one-way analysis of variance, using GraphPad v5.0 software (GraphPad Software, Inc., La Jolla, CA, USA). P<0.05 was considered to indicate a statistically significant difference.

## Results

### Preliminary phytochemical screening of total saponins

In order to determine the existence of saponins among the four fractions of the *A. julibrissin* extract, traditional preliminary phytochemical screening assays (foam and hemolytic tests) were conducted. The majority of saponins were contained within 70% E (named TSAJ).

### TSAJ inhibits VEGF-induced viability of endothelial cells

**Figure 2:**
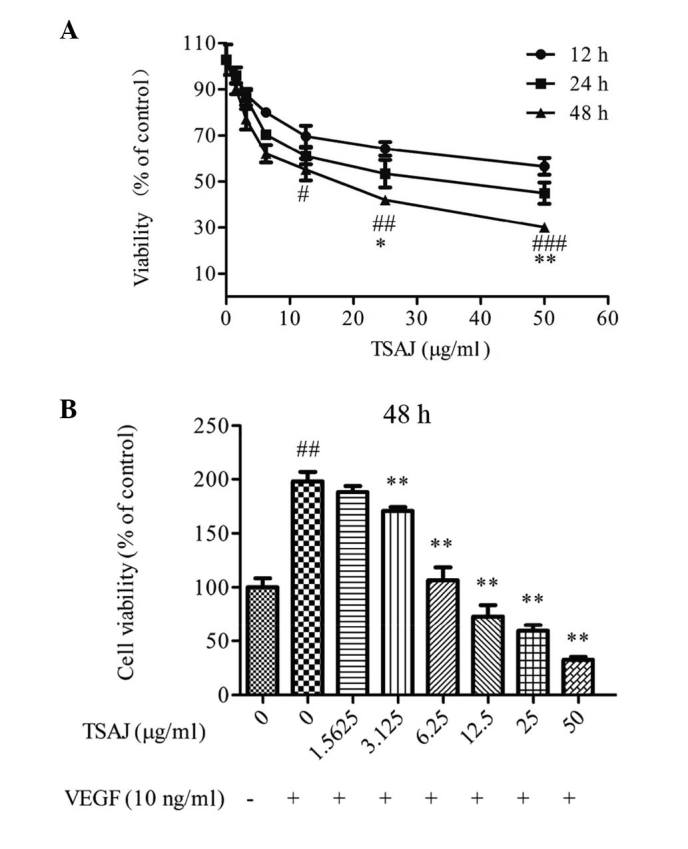
Effects of total saponins from *Albizia julibrissin* (TSAJ) on cell viability. (A) TSAJ inhibited the viability of Ea.hy926 endothelial cells maintained in normal culture conditions. ^#^P<0.05, ^##^P<0.01, ^###^P<0.001, 48 h-treated group vs. 12 h-treated group; ^*^P<0.05, ^**^P<0.01, 48 h-treated group vs. 24 h-treated group. (B) TSAJ inhibited vascular endothelial growth factor (VEGF)-induced increases in cell viability in a dose-dependent manner. Results are represented as the percentage of vehicle-treated control, and the data represent the mean ± standard error of the mean. ^##^P<0.01, the VEGF-treated group vs. the untreated group; ^**^P<0.01, the VEGF and TSAJ-treated group vs. the VEGF-treated group.

The inhibitory effects of TSAJ on cell viability in normal growth medium (containing 10% FBS) was initially evaluated using a CCK-8 assay. TSAJ inhibited cell viability in a dose- and time-dependent manner (Fig. 2A). When the Ea.hy926 cells were treated with 18 μg/ml TSAJ for 48 h, a significant inhibitory effect on viability was observed.

The present study also determined whether TSAJ inhibited VEGF-induced endothelial cell viability, at the same dose range. Stimulation with VEGF for 48 h increased the number of Ea.hy926 cells by ~2 fold (Fig. 2B). Treatment with TSAJ significantly suppressed the VEGF-induced increase in cell viability at doses from 9 μg/ml, in a dose-dependent manner. These results demonstrate that VEGF-activated endothelial cells were more sensitive to TSAJ, as compared with those cultured in normal growth medium, and that TSAJ may be a potent inhibitor of VEGF-induced increases in endothelial cell viability.

### TSAJ inhibits VEGF-induced migration of endothelial cells

**Figure 3:**
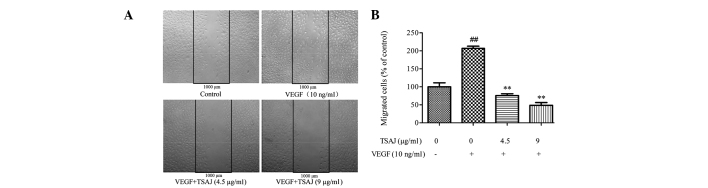
Effects of total saponins of *Albizia julibrissin (*TSAJ) on vascular endothelial growth factor (VEGF)-induced migration of endothelial cells (wound-healing assay). (A) Representative photomicrographs of Ea.hy926 cell migration (magnification, ×40). (B) Number of migrated cells following treatment with the various processing methods for 14 h. Results are represented as percentage of the vehicle-treated control, and the data represent the mean ± standard error of the mean. ^##^P<0.01, the VEGF-treated group vs. the untreated group; ^**^P<0.01, the VEGF and TSAJ-treated group vs. the VEGF-treated group.

During angiogenesis, endothelial cell migration is an essential process (26). Therefore, the effects of TSAJ on the migratory ability of Ea.hy926 cells was determined using a wound-healing assay. VEGF significantly stimulated the directional migration of endothelial cells (Fig. 3A), and the number of migratory cells in the VEGF-treated group was >2 times more, as compared with the control group (Fig. 3B). The migratory rate of the TSAJ-treated endothelial cells was markedly suppressed, as compared with the VEGF-treated cells, and the inhibitory effects were more obvious at 9 μg/ml, as compared with at 4.5 μg/ml. These results suggest that TSAJ significantly suppressed VEGF-induced migration of endothelial cells in a dose-dependent manner.

### TSAJ inhibits VEGF-induced endothelial cell tube formation

**Figure 4:**
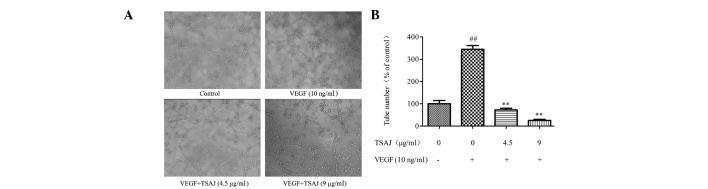
Effects of total saponins of *Albizia julibrissin* (TSAJ) on vascular endothelial growth factor (VEGF)-induced endothelial cell tube formation *in vitro*. (A) Representative photomicrographs of Ea.hy926 endothelial cell tube formation on growth factor-reduced two-dimensional Matrigel™ (magnification, ×40). (B) Graph shows the quantitative effects of TSAJ on VEGF-induced tube formation. Results are represented as the percentage of vehicle-treated control, and the data represent the mean ± standard error of the mean. ^##^P<0.01, the VEGF-treated group vs. the untreated group; ^**^P<0.01, the VEGF and TSAJ-treated group vs. the VEGF-treated group.

Angiogenesis is the formation of blood vessels from pre-existing vasculature, and is considered to be a complex process. During angiogenesis, the maturation of migrated endothelial cells into a capillary tube is a critical early step (27). Therefore, the present study investigated how TSAJ regulates capillary tube formation. Treatment with VEGF alone stimulated complete and robust tubular capillary structures (Fig. 4A). However, treatment with TSAJ massively disrupted the capillary tube network, and the tubes formed in the TSAJ-treated group were rather incomplete. In addition, the number of tubes formed was quantified using Image-Pro Plus software in low power field, and tube formation was shown to be negatively interrupted in response to treatment with TSAJ (Fig. 4B). These results demonstrate that TSAJ effectively inhibits VEGF-induced Ea.hy926 cell tube formation on Matrigel™ in a dose-dependent manner.

### TSAJ inhibits activation of the VEGFR2-mediated signaling pathway

**Figure 5:**
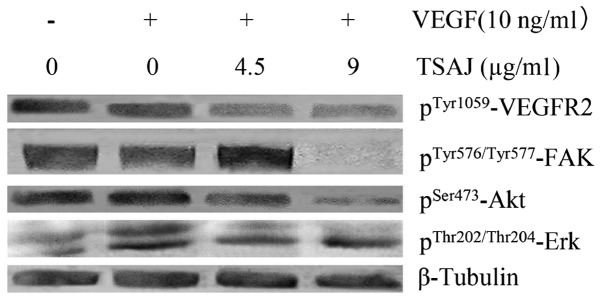
Effects of total saponins of *Albizia julibrissin* (TSAJ) on vascular endothelial growth factor (VEGF)-induced angiogenesis in Ea.hy926 endothelial cells. Representative western blot analysis for phosphorylated-(p-)VEGF receptor 2 (VEGFR2), p-focal adhesion kinase (Fak), p-Akt and p-extracellular signal-regulated kinase (p-Erk), all of which were markedly inhibited by TSAJ. β-Tubulin was used as a loading control.

It is well known that the VEGF signaling pathway has a vital role in angiogenesis. VEGF binding to VEGFR2 results in the autophosphorylation of VEGFR2, which subsequently activates various downstream signaling molecules that are responsible for endothelial cell migration, proliferation and survival (28). To further elaborate the underlying mechanisms of the anti-angiogenic effects of TSAJ, the present study evaluated some key signaling molecules involved in the VEGFR2-mediated signaling pathway. VEGF induces survival of endothelial cells mainly through activation of Akt, whereas activation of Erk1/2 mitogen-activated protein kinases (MAPKs) is thought to be essential for VEGF-induced proliferation. VEGF-induced phosphorylation of VEGFR2 at Tyr-1175 was suppressed by treatment with TSAJ in a dose-dependent manner (Fig. 5). In addition, TSAJ significantly suppressed the VEGF-induced phosphorylations of Akt (Ser^473^) and Erk (Thr^202^/Thr^204^). The effects of TSAJ on the phosphorylation of FAK were also examined, and TSAJ was shown to inhibit VEGF-induced phosphorylation of FAK, when administered at a dose of 9 μg/ml. These results indicate that TSAJ exerts its anti-angiogenic effects by selectively targeting certain signaling events downstream of VEGFR2.

### TSAJ inhibits VEGF-induced blood vessel formation in mice

**Figure 6:**
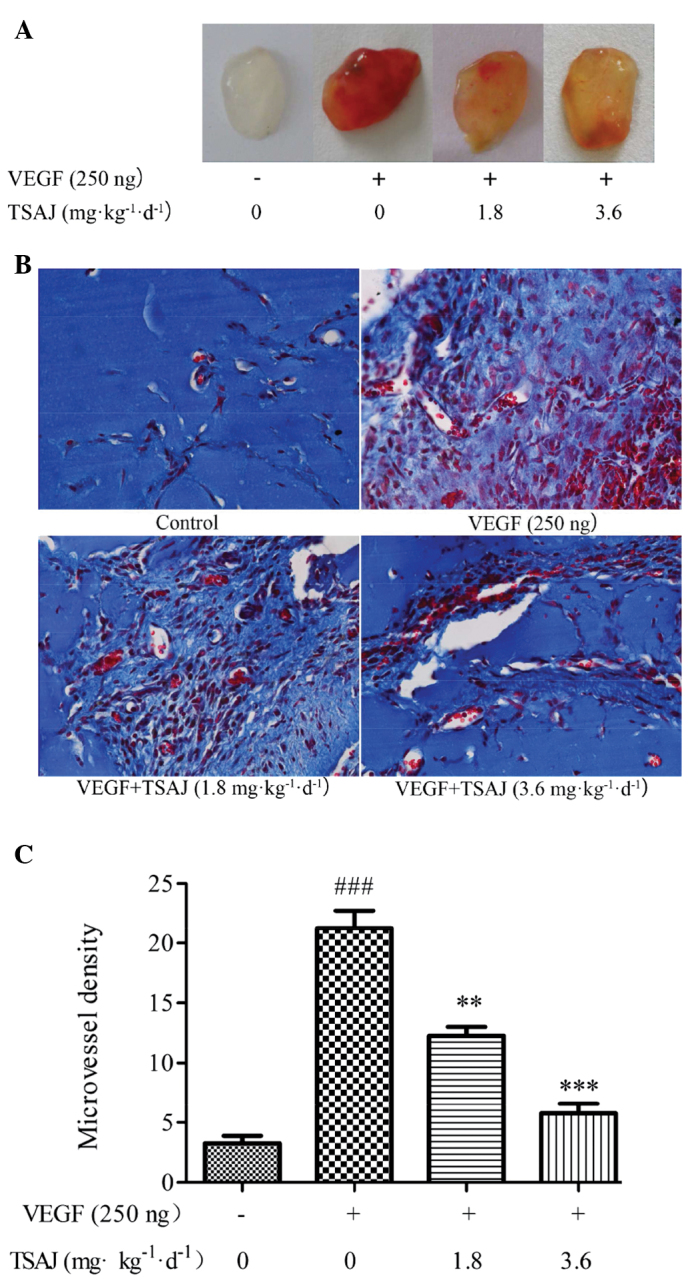
Anti-angiogenic effects of total saponins of *Albizia julibrissin* (TSAJ) on a murine Matrigel™ plug assay (n=6). (A) TSAJ inhibited angiogenesis induced by vascular endothelial growth factor (VEGF) in the murine Matrigel™ plug model. (B) Representative Masson’s Trichrome (M-T) staining of Matrigel™ plugs from each group. (magnification, ×400). (C) Degree of angiogenesis was determined directly by M-T staining assay. Data are expressed as the mean ± standard error of the mean. ^###^P<0.001, the VEGF-treated group vs. the untreated group; ^**^P<0.01 and ^***^P<0.001, the VEGF and TSAJ-treated group vs. the VEGF-treated group.

The results of the present study demonstrated that TSAJ effectively inhibited VEGF-induced angiogenesis *in vitro*, therefore the anti-angiogenic effects of TSAJ were subsequently validated *in vivo*. The anti-angiogenic effects of TSAJ were evaluated in a mouse Matrigel™ plug model, which is a powerful *in vivo* angiogenesis assay (29). Matrigel™ plugs containing VEGF alone were dark red, whereas the Matrigel™ plugs of the TSAJ-treated group were light red (Fig. 6A). Conversely, the Matrigel™ plugs of the vehicle group were avascular and pale white. These superficial phenomena suggest that abundant functional vasculatures had formed within the VEGF group, whereas fewer blood vessels had formed within the Matrigel™ plugs of the TSAJ-treated group. To provide further evidence for this finding, the number of functional vessels within the Matrigel™ plugs were compared by M-T staining, which stained the Matrigel™ blue and the endothelial cells/vessels red (Fig. 6B). Fewer vessels were observed in the Matrigel™ plugs of the mice treated with VEGF and TSAJ, as compared with those in the mice treated with VEGF alone (Fig. 6C). These results demonstrate that TSAJ strongly suppresses VEGF-induced angiogenesis 14 days after Matrigel™ implantation. These findings were concordant with the results of the *in vitro* tube formation assay.

### Anti-tumor effects of TSAJ on a H22 hepatoma cell transplantation model

Angiogenesis is the pivotal step in tumor growth and metastasis, which provides necessary oxygen and nutrients for the tumor (30). To investigate the anti-tumor effects of TSAJ, a H22 hepatoma tumor model was generated.

**Figure 7:**
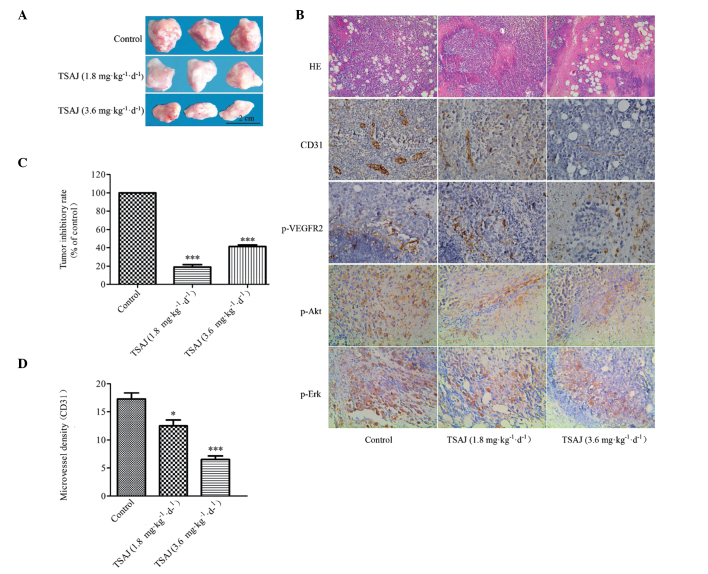
Effects of total saponins of *Albizia julibrissin* (TSAJ) on a H22 hepatoma cell transplantation model. (A) Representative images of solid tumor tissues following treatment for 14 days. (B) Serial sections (4 μm) were processed for hematoxylin and eosin (HE) staining (magnification, ×100), and immunohistochemistry (magnification, ×400) with antibodies targeting cluster of differentiation 31 (CD31), phosphorylated-vascular endothelial growth factor receptor 2 (p-VEGFR2), p-Akt and p-extracellular signal-regulated kinase (Erk). (C) Tumor mass was weighed and the tumor inhibitory rate was calculated. (D) Results of microvessel density from H22 hepatoma tissue. Data are expressed as the mean ± standard error of the mean from three independent experiments. ^*^P<0.05 and ^***^P<0.001 vs. the control group.

Tumor growth was significantly suppressed by treatment with TSAJ, at the dose of 3.6 mg/kg/d, after 14 days of treatment (Fig. 7A). The tumors of the TSAJ-treated group were significantly smaller, as compared with those in the control group. The average tumor weight in the control group was 2.023±0.119 g, whereas the average tumor weight in the 3.6 and 1.8 mg/kg/d TSAJ-treated groups were 1.189±0.106 g and 1.637±0.167 g, respectively. The pathological results showed that extensive densely vesicular nuclei and sparse cytoplasm was observed in the TSAJ-treated groups. Furthermore, as the concentration of TSAJ increased, large areas of necrosis appeared in the tumor tissue, and the region of necrosis expanded (Fig. 7B). The inhibitory rate of tumors in the 3.6 mg/kg/d treatment group was 41.245% (Fig. 7C). These results indicate that the rate of proliferation of tumor cells was inhibited by TSAJ, in a dose-dependent manner.

Although tumor growth was indeed suppressed in response to treatment with TSAJ, the anti-tumor mechanisms of TSAJ still required further validation. To investigate new blood vessel formation in solid tumors, immunostaining for CD31 was performed (Fig. 7B). Microvessels were stained brown by CD31, which is a specific-endothelial marker. The MVD values in the TSAJ-treated groups were significantly reduced, as compared with the control group (Fig. 7D). Furthermore, treatment with TSAJ significantly decreased the expression levels of p-VEGFR-2, p-Erk and p-Akt (brown-stained field), as compared with the control group (Fig. 7B). These results suggest that TSAJ is a potent angiogenic inhibitor, which inhibits tumor neovascularization and suppresses tumor growth through inhibition of the VEGFR-2 signaling pathway.

## Discussion

In recent years, numerous efforts have been made to identify potential anti-angiogenic agents from traditional Chinese medicinal herbs. AJ has been used to treat various diseases for thousands of years (31); however, little is currently known with regard to the inhibitory effects of AJ on angiogenesis. Previous studies have reported that the chemical composition of AJ includes saponins, flavonoids, alkaloids and polysaccharides (32), and saponins are considered to be the main anti-tumor component (33). The present study explored the anti-angiogenic mechanisms of TSAJ. Angiogenesis depends on the proliferation, migration and tube formation of endothelial cells (34), and VEGF is a major proangiogenenic factor that mediates these processes (35). The results of the present study demonstrated that VEGF significantly promoted the proliferation of Ea.hy926 cells. However, treatment with TSAJ resulted in a marked decrease in the VEGF-induced proliferation of Ea.hy926 cells, in a dose-dependent manner. Migration of endothelial cells as a response to pro-angiogenic factors is an integral feature of angiogenesis. The inhibitory effects of TSAJ *in vitro* were evaluated using a wound healing assay and a growth factor-reduced two-dimensional Matrigel™ model. In the two models, TSAJ inhibited the VEGF-induced cell migration and capillary tube formation of Ea.hy926 cells, thus suggesting that treatment with TSAJ suppressed VEGF-induced angiogenesis by restraining the cellular connection.

Angiogenesis stimulated by VEGF is a complex process. Upon binding to VEGF, VEGFR2 undergoes autophosphorylation and transmits the signal to downstream molecules associated with angiogenic processes. The PI3K/Akt and RAF/MEK-ERK pathways are involved in VEGF-induced angiogenesis. The phosphorylation of VEGFR2 at Tyr-1175 is required for activation of Akt, which contributes to the proliferation and survival of endothelial cells (36). In addition, phosphorylation of VEGFR2 at Tyr-1175 activates the MAPK/ERK cascades, which also promote the proliferation of endothelial cells (36). FAK and its substrate paxillin are signaling molecules associated with VEGF-induced migration, which participate in focal adhesion during cell migration (37–39).

To elucidate the underlying anti-angiogenic mechanisms of TSAJ, the expression of signaling molecules and pathways were analyzed by western blotting. Treatment with TSAJ suppressed the phosphorylation of VEGFR2 at the Tyr1175 site in endothelial cells, suggesting that TSAJ may inhibit the kinase activity of VEGFR2 by downregulating the expression of VEGFR2. Furthermore, TSAJ significantly suppressed the VEGF-induced phosphorylation of Akt (Ser473) and Erk (Thr202/Thr204), in a dose-dependent manner. However, the phosphorylation of FAK was only markedly inhibited when the cells were treated with TSAJ at a dose of 9 μg/ml. These results suggest that TSAJ modulated VEGF-induced vascular permeability and angiogenesis by inhibiting the phosphorylation of VEGFR2, FAK, AKT, and ERK in endothelial cells.

The results of the present study demonstrated that TSAJ exhibits highly anti-angiogenic effects *in vitro*. In order to confirm the anti-angiogenic efficacy of TSAJ in an animal model, a mouse Matrigel™ plug assay was used. The Matrigel™ plug assay is a relatively quick and easy method to evaluate angiogenic and anti-angiogenic compounds (25). TSAJ markedly suppressed VEGF-induced neovascularization in the Matrigel™ plug assay, by inhibiting endothelial cell infiltration into the plug. The number of functional capillaries containing erythrocytes were markedly decreased in the TSAJ-treated group, as compared with the VEGF-induced group. These results suggest that TSAJ inhibited VEGF-induced neovascularization in the Matrigel™ plug assay, by inhibiting the invasion of endothelial cells.

Since the anti-angiogenic effects of TSAJ *in vivo* were validated using the Matrigel™ plug model, the H22 hepatoma tumor model was used to provide further evidence regarding the inhibitory effects. The hepatoma tumor model is widely used in the field of anti-tumor research (40). In the primary stages of tumor growth, tumor cells rapidly proliferate, and an abundance of nutrients and oxygen is required (41). Therefore, oxygen and nutrients, which are provided by tumor blood vessels, are considered to be critical factors to limit the growth of tumors. Once the formation of blood vessels is suppressed, tumor cells will undergo necrosis, due to ischemia and hypoxia (42,43). In response to hypoxia, vasculogenesis is induced by VEGF-A165, which is secreted by tumor cells and can improve the tumor microenvironment (7). Based on this concept, inhibition of angiogenesis may be an effective approach to prevent and treat cancer. In the present study, tumor growth was significantly inhibited in the TSAJ-treated group. Furthermore, the percentage of necrosis in the TSAJ-treated group was higher, as compared with that of the control group. Immunohistochemical staining with antibodies targeting CD31 demonstrated that the MVD was significantly decreased in the TSAJ-treated group, which limited the blood supply to the tumor. In addition, immunohistochemical staining showed that the expression of p-VEGFR2, p-Akt and p-Erk were significantly suppressed in the TSAJ-treated group. These signaling molecules are the main factors associated with the VEGF/VEGFR2 signaling pathway in angiogenesis of vascular endothelial cells. Therefore, downregulation of p-VEGFR2, p-Akt and p-Erk expression may be the underlying mechanism for the anti-angiogenic effects of TSAJ.

In conclusion, the present study was the first, to the best of our knowledge, to demonstrate that TSAJ is able to inhibit VEGF-induced endothelial cell proliferation, migration, and tube formation, by suppressing the activation of VEGFR2 and its downstream signaling of Fak, Akt and Erk *in vitro* and *in vivo*. Furthermore, the anti-angiogenic effects of TSAJ *in vivo* demonstrated that TSAJ may be considered as a potential oral anti-angiogenic drug, which targets the VEGF/VEGFR2 signal pathway.

## Abbreviations

AJ: *Albizia julibrissin*
TSAJ: total saponins from *Albizia julibrissin*
FBS: fetal bovine serum
HE: hematoxylin and eosin
M-T: Masson’s Trichrome
IHC: immunohistochemistry
TBST: Tris-buffered saline with Tween
VEGF: vascular endothelial growth factor
p-VEGFR2: phosphorylated-vascular endothelial growth factor receptor 2
p-Erk: phosphorylated-extracellular signal-regulated kinase
p-Akt: phosphorylated-Akt
p-Fak: phosphorylated focal adhesion kinase
